# Effects of a theory-based training program with follow-up home visits on self-management behavior, glycemic index, and quality of life among Iranian patients with type 2 diabetes mellitus

**DOI:** 10.1186/s12889-022-13959-3

**Published:** 2022-08-16

**Authors:** Mohammad Hossein Kaveh, Maryam Montazer, Masoud Karimi, Jafar Hassanzadeh

**Affiliations:** 1 Research Center for Health Sciences, Institute of Health, School of Health, Shiraz University of Medical Sciences, Razi Boulevard, Shiraz, P.O. Box 71536-75541, Iran; 2 Department of Health Promotion, School of Health, Shiraz University of Medical Sciences, Shiraz, P.O. Box 71536-75541, Iran; 3 Department of Epidemiology, School of Health, Shiraz University of Medical Sciences, Shiraz, P.O. Box 71536-75541, Iran

**Keywords:** Type 2 diabetes mellitus, Social cognitive theory, Self-management, Home visit, Quality of life

## Abstract

**Background:**

Uncontrolled diabetes is an important public health problem that endangers the quality of life of patients. Promoting self-management through well-planned training is an essential strategy to control diabetes effectively. This study aimed to examine the effects of a training program based on social cognitive theory (SCT) on self-management behavior, glycemic index, and quality of life among patients with type 2 diabetes mellitus.

**Methods:**

This is a quasi-experimental study with a pretest–posttest design. The statistical population included 106 adults with type 2 diabetes mellitus assigned to the intervention and control groups [n_1_ = n_2_ = 53], who received services from two urban health centers. A multi-method, SCT-based training program consisting of six 60–80-min sessions was run, followed by 2–3 follow-up home visits [once a month for each participant] for the intervention group. The data were collected before and three months after the intervention and were analyzed in SPSS 19.

**Results:**

Before the intervention, there was no significant difference between the two groups regarding the main variables. After the intervention, there was a significant increase in the intervention group's mean scores of knowledge, self-efficacy, social support, outcome expectations, self-regulation, self-management behavior, glycemic index, and quality of life. There were no significant changes in these constructs in the control group after the intervention. The regression analysis results indicated that social cognitive theory and self-management could explain the variance in quality of life [adjusted R-squared = 0.476].

**Conclusions:**

The findings support the effectiveness of the multi-method, SCT-based educational intervention in improving self-management behaviors, glycemic index, and quality of life among patients with type 2 diabetes mellitus. It is suggested that the quality of type 2 diabetes care programs should be promoted. However, further research is needed to evaluate the long-term outcomes.

## Introduction

Diabetes mellitus, one of the most common chronic non-communicable diseases, is the leading cause of life-threatening disabilities, costly complications, decreased quality of life, and diminished life expectancy [1–3]. It substantially burdens community health and socioeconomic development [4].

A high proportion of cases of diabetes are uncontrolled. The global prevalence of uncontrolled diabetes is reportedly 40% to 60% [5]. In this context, 36% of patients with type 2 diabetes mellitus (T2DM) in developing countries have never measured their hemoglobin A1c [HbA1c], and only 36.4% of those who have measured HbA1c have optimal glycemic control [6]. In Iran, the prevalence of uncontrolled T2DM is approximately 60%, consistent with studies carried out in the Middle East [7]. Uncontrolled T2DM is associated with several complications, such as glaucoma and cataracts, foot problems, skin infections, urinary tract infections, and genital problems. It has also been associated with cardiovascular complications, such as heart attack, renal failure, vision loss, peripheral artery disease, and amputation [8]. Diabetes is a major risk factor for premature death, mainly due to cardiovascular and cerebrovascular diseases [9]. In addition, it increases the risk of contracting infectious diseases, such as COVID-19, and the chance of adverse clinical outcomes [10].

Effective diabetes management includes three key components: medical treatment, environmental support [especially from the family], and self-management. Self-management is vital in accessing and benefiting from the other two. Numerous factors affect the performance and adherence to self-management behaviors at multiple levels. Many studies address the lack of knowledge and social and organizational [health centers] support, inappropriate attitudes, inadequate self-efficacy, and poor self-regulatory skills [11–14]. Despite the multifactorial nature of self-management behaviors, most interventions focus solely on increasing knowledge and attitudes, lacking follow-ups, and neglecting contextual conditions, including socioeconomic status and social support required by the family [15–18].

The design of educational interventions to cause desirable behavioral changes improves when all factors related to target behavior[s] determinants are considered [19]. Behavior change theories help give a better insight into the factors that influence behavior in the target population and choose the right approach to designing, implementing, and evaluating the intervention [19–21].

Considering the intrapersonal and interpersonal factors affecting self-management among diabetic patients, the social cognitive theory (SCT), developed by Albert Bandura, was found to be a suitable theoretical framework to conduct and evaluate the intervention in this study. Its underlying concept, based on which human behavior is explained, is the triadic reciprocal determinism or the dynamic interplay among personal cognitive factors, the environment, and behavior [19, 20].

SCT defines several constructs as individual and interpersonal determinants of behavior. In the following, the constructs identified in the present analysis of health behavior in the target population are briefly described:

Badura states that knowledge is a precondition for behavior change [20]. Knowledge is a collection of information, facts, and experiences acquired by a person during life and education [21]. It enables recognizing the health risks and benefits of health-related choices and is a guide for choosing information and actions beneficial to health [19]. Outcome expectations refer to predicting possible outcomes that will result from the desired behavior [expected results] [22]. Self-efficacy refers to an individual's self-confidence in their ability to perform the behavior that leads to the result. Self-regulation refers to controlling behaviors based on personal standards [20]. Finally, social support implies understanding the support and encouragement received from one's social network [19].

As a practical implication of social support, home-based visits and/or follow-ups may help patients with T2DM improve self-management as an integral strategy [23]. The overcrowding of outpatient care centers and the mobility-related difficulties that T2DM patients face limit the provision of adequate training and follow-up care. Home visits are good opportunities to further educate patients and family members, involve them in the care process, and develop social support for patients [24]. This strategy can enhance adherence to self-care and improve health outcomes and quality of life [23]. Research shows that pursuing care through home visits promotes glycemic control, improves the quality of life [25], increases patient confidence in decision making, and enhances self-care behaviors [26].

In support of using SCT as a theoretical framework, evidence suggests that treatment plans, self-efficacy, situational factors, social support, and the involvement of significant others are decisive in diabetic self-management behavior [27, 28]. In addition, a cross-sectional study in Iran shows that SCT’s constructs significantly predict self-care behavior among T2DM patients [16]. It has been demonstrated that social support, self-efficacy, self-regulation, and outcome expectations are important determinants of nutritional behavior [14]. However, there are conflicting findings on the relationship between such variables and some outcomes in patients with type 1 diabetes (T1D): While high self-efficacy has been associated with lower hemoglobin A1c levels [29], no significant relationship has been found in another relevant study [30].

No relevant study has been found with a theoretical framework similar to the present study. Hence, the present study was designed to examine the effects of an SCT-based training program with follow-up home visits on SCT constructs, self-management behavior, glycemic index, and quality of life among patients with T2DM.

## Methods

### Design

This was an interventional study using a pretest–posttest control group design.

### Participants and sampling

The statistical population included patients with T2DM covered by two urban health centers affiliated with Jundishapur University of Medical Sciences in Ahvaz, the largest city in Southwestern Iran. Based on a previous study [21], where α = 0.05, 1-β = 0.80, SD_1_ = 1.48, SD_2_ = 2.11, d = ∆ = 1.04, and a 10% probable drop, 53 participants were assigned to each group. The following formula was used to calculate the sample size [31]:

$$n=\frac{\left(\sigma \begin{array}{c}2\\ 1\end{array}+\sigma \begin{array}{c}2\\ 2\end{array}\right){\left({\mathcal{z}}_{1-a}+{\mathcal{z}}_{1-\beta }\right)}^{2}}{{\Delta }^{2}}$$

The participants were selected using two-stage cluster random sampling. First, out of 21 community health centers, two were selected by simple random sampling. These two centers [as clusters] were randomly assigned to the intervention and control groups. A list of patients with uncontrolled T2DM [HbA1C level ≥ 6.5%, as one of the main inclusion criteria] was prepared using the Electronic Population Health Portal. From this list, 53 patients were selected using the systematic random sampling method. The selected individuals were screened based on the inclusion criteria. In cases where criteria were not met, the eligible ones were replaced with the previous or next patient from the list. The selected patients were contacted by telephone, briefed on the project, and invited to attend the health center if they agreed to participate. They were given more information about the research project at the health center, including ethical considerations. Finally, they were asked to sign an informed consent form if they agreed to participate.

The inclusion criteria included individuals in the 30–60 age range, not suffering from any mental or psychological disorders, having elementary school education at a minimum, a definite diagnosis of T2DM in the last six months, HbA1C level ≥ 6.5%, not suffering from Type 1 diabetes, having access to a telephone or mobile phone and the ability to use it efficiently for the follow-up, completing the informed consent form, no prior history of participating in other diabetes education programs, and not suffering from chronic diseases (cancer and severe hypertension, or serious cognitive problems) and health problems affecting diabetes self-management behaviors (end-stage renal disease, vision loss, and cardiac insufficiency). The exclusion criteria included cognitive impairment and inability to communicate (based on the information in the electronic health record), pregnancy, and occurrence of illnesses or physical problems (potentially) influencing self-care behaviors.

### Intervention

**Fig. 1:**
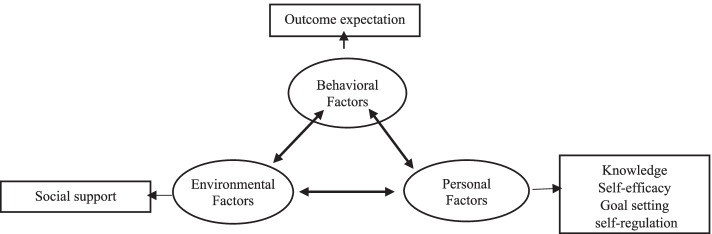
Constructs of the social cognitive theory

The educational intervention was implemented through six sessions of 60–80 min for each intervention subgroup. The participants were divided into four subgroups [*n* = 12–15] to facilitate the implementation of participatory learning methods. The SCT was used as the guiding framework in designing and implementing the intervention (Fig. 1).

Educational content included knowing about T2DM, complications of diabetes if left uncontrolled, elements of effective diabetes control [nutrition, physical activity, medication adherence], self-management responsibility, and environmental support [32]. More details of the curriculum based on SCT’s constructs are presented in Table 1.

**Table 1:** Educational content based on constructs of SCT

Constructs	Education details
Knowledge	The interactive lecture techniques used included presentations, video clips, booklets, and a replica of the gastrointestinal tract to improve knowledge of diabetes
Self-efficacy	Four methods recommended by Bandura [19] were used to improve patients’ self-efficacy for doing self-management behaviors. For example, to promote physical activity, simple and short activities, such as walking, were started [mastery experiences], and physically active people were used as role models [observational learning]. Besides, relaxation exercises, such as yoga, were used to improve the participants’ emotional states. The exercise instructor and one of the researchers attended the training sessions [verbal persuasion and reducing stress [19, 22]
Goal setting and self-monitoring	The participants were asked to evaluate their performance concerning each self-management component individually and then in small groups to improve their self-regulatory skills [self-monitoring]. Appropriate goals and step-by-step plans were set to improve their performance through partnerships with group members [goal-setting]; they monitored their performance and gave feedback to themselves and each other. The feedback was also given through face-to-face conversations and telephone or WhatsApp calls. In addition, the patients’ progress was rewarded [self-reward]. The participants were advised to refer to the introduced booklets and resources [self-instruction] to promote their learning [20]
Outcome expectations	The benefits and barriers of self-care behaviors were identified using small group techniques such as snowballs and buzzing [33]. Discussion and question–answer methods were used to improve outcome expectations. Additionally, evidence supporting the positive outcomes of self-management behaviors was presented through mini-lectures. Moreover, the individuals who successfully controlled their type 2 diabetes were asked to share their experiences with group members
Environmental factors	During the project, efforts were made to provide social support from multiple sources, including family, health care providers, and peers [19]. During home visits, family members were briefly trained and encouraged to support their patients as needed. A nutritionist provided nutrition counseling in some training sessions. In addition, a member of the research team [second author] and a trained health worker accompanied the patients during the exercise sessions and were on-call to respond to patients by phone and WhatsApp

At the end of the training program, each participant conducted at least two home visits once a month, during which they were provided with additional information. Mini-lectures and pamphlets were used to increase family members' information and engage them in patient care. They were assisted in monitoring their blood sugar and self-care behaviors. Furthermore, they were given feedback to improve their diabetes self-management. It should be noted that patients in the control group experienced routine programs in health centers. However, they were provided with the training materials after completing the project due to ethical considerations.

### Measuring tools

A four-part questionnaire was used to collect the required data. The data included the demographics, SCT constructs, self-management behaviors, and quality of life at baseline and three months after completion of the educational intervention. In addition, HbA1c and fasting blood sugar (FBS) were measured at baseline and three months after the intervention. More details of these measurements are given below.

#### Demographic form

Demographic variables including age, sex, employment status, literacy levels, homeownership status, ethnicity, and duration of diabetes since diagnosis were measured using forced-choice questions.

#### The SCT-based questionnaire

The administered SCT questionnaire was developed using relevant scientific sources [19, 20, 22]. The content validity of this tool was evaluated qualitatively by a panel of experts (*n* = 10) and quantitatively determined by calculating the content validity ratio (CVR) and content validity index (CVI) [34, 35]. The means of CVR and CVI were 0.88 and 0.96, respectively. The questionnaire's reliability was confirmed in a pilot study conducted on a sample of patients (*n* = 20) from the study population. The Cronbach alpha coefficients of the SCT constructs ranged from 0.72 to 0.98. The measuring and scoring methods of each construct are stated in the following. In general, higher scores indicate the better status of each construct.

### Knowledge

The diabetes-related knowledge was measured using a true/false test with nineteen questions. Correct and incorrect answers were scored 1 and 0, respectively. Cronbach’s alpha coefficient was 0.98.

### Self-efficacy

The 5-point Likert-type questionnaire used to measure self-efficacy comprised ten statements. For example, "how confident are you to maintain your diet when eating with people without diabetes?" Responses were rated from 1 for "very low" to 5 for "very high." Cronbach’s alpha was 0.80.

### Social support

Perceived social support was measured using seven statements, including "not at all," "rarely," "sometimes," "most of the time," and "always." For example, "my family accompanies and helps me to do exercise." The responses were scored from 1 for "not at all" to 5 for "always." The total score of this construct ranged from 7 to 35. The alpha coefficient was 0.89.

### Self-regulation

Eleven questions with a 5-point Likert scale (completely disagree, disagree, have no opinion, agree, completely agree) were used to measure self-regulation skills. For example, "I can make a workable plan to achieve my diabetes control goals." Answers were scored as 1 to 5, respectively. Cronbach alpha coefficient for this construct was 0.90.

### Outcome expectations

Outcome expectations were measured and scored using 14 items on a 5-point Likert scale (1 = completely disagree, 2 = disagree, 3 = have no opinion, 4 = agree, and 5 = completely agree). For example, one of the items was, "By engaging in self-care behaviors, I can substantially prevent the complications of diabetes, such as blindness, renal disease, diabetic foot, and cardiovascular disease." The Cronbach’s alpha coefficient was 0.72.

### Self-management behavior

The domains of diabetes self-management behaviors included diet, physical activity, and adherence to a medical care plan, such as regular visits to the physician, measuring blood sugar, and taking medication as prescribed. The participants' behaviors were measured and accordingly scored using twenty-five questions on a 3-point Likert scale (1 = requires correction, 2 = acceptable, 3 = satisfactory).

### Diabetes quality of life-brief clinical inventory

The Diabetes Quality of Life Brief Clinical Inventory (DQOL-BCI) was used to evaluate the quality of life of patients. The feasibility, reliability, and validity of the Persian version of DQOL-BCI were confirmed in 2011 [35]. This tool consists of fifteen items responded to via a five-point Likert scale. For some questions (e.g., "how often do you get insomnia due to diabetes?"), responses were rated from 1 for "never" to 5 for "always." For some other questions (e.g., "how satisfied are you with your current diabetes treatment status"), answers were rated 1 for "completely disagree" to 5 for "completely agree." The internal consistency of this instrument was also confirmed in the pilot study [alpha = 0.97].

### Statistical analysis

The data were analyzed in SPSS (Ver. 19). The Kolmogorov–Smirnov (KS) test was used to examine the normal distribution of the variables. The result showed normal distribution for some but not all variables. Regarding the central limit theorem [34] and the sample size of more than 30 participants in each group, parametric statistical tests were used to analyze the data. Therefore, the paired t-test was used to compare the pre-post intervention within-group means. An independent t-test was used to compare the mean scores of study constructs between groups. Furthermore, a multivariate regression analysis was conducted to predict the quality of life based on the main study constructs.

## Results

The mean age in the intervention and control groups was 49.32 ± 7.05 and 49.73 ± 7.95 years, respectively (t = 0.275, df = 97, *p* = 0.784). The majority of the participants in the groups were female [65.7%], housekeepers [58.6%], married [88%], and their education levels ranged from the 6^th^ to the 12^th^ grade. Besides, the majority of the participants were from Arab tribes (74.7%), homeowners (70.7%), and lived with their spouses and children (87.9%). The groups displayed no statistically significant differences in terms of demographic variables. Seventy-seven patients (77.8%) had had diabetes for more than five years. The mean duration of T2DM since diagnosis was not statistically significant between the intervention (2.60 ± 0.72 years) and control (2.73 ± 0.60 years) groups (t = 1, df = 97, *p* = 0.32).

At baseline, the mean score of knowledge in the intervention (13.58 ± 2.71) and control (13.20 ± 2.91) groups was not statistically different. After the intervention, the mean score of knowledge was 18.54 ± 0.58 in the intervention group and 13.34 ± 2.81 in the control group, and the independent samples t-test showed significant differences between the two groups (t = 12.76, df = 97, *p* < 0.001). The independent t-test indicated that the mean changes in the knowledge score in the intervention group (4.96 ± 0.37) and control group (0.14 ± 0.10) were significantly different (Table 2).

**Table 2:** Comparison of mean scores of SCT’s constructs within and between intervention and control groups, before and after the training program

Variable	Group	Before		After		Sig.*	Difference	
		Mean	SE	Mean	SE		Mean	SE
Knowledge	Intervention	13.58	0.38	18.54	0.08	< 0.001	4.96	0.05
	Control	13.20	0.41	13.34	0.40	0.16	0.14	0.01
Sig.**		0.50		< 0.001		––––	< 0.001	
Outcome expectation	Intervention	46.14	0.90	57.02	0.64	< 0.001	10.88	0.58
	Control	46.55	0.78	46.89	0.77	0.74	0.34	1.06
Sig.**		0.73		< 0.001		––––	< 0.001	
Self-efficacy	Intervention	29.54	0.34	36.70	0.41	< 0.001	7.16	0.09
	Control	28.57	0.92	28.59	0.97	0.23	0.02	0.07
Sig.**		0.59		< 0.001		––––	< 0.001	
Social support	Intervention	15.62	0.63	28.86	0.41	< 0.001	13.24	0.62
	Control	16.02	0.39	16.24	0.41	0.23	0.22	0.18
Sig.**		0.59		< 0.001		––––	< 0.001	
Self-regulation	Intervention	30.76	0.61	42.72	0.54	< 0.001	11.96	0.73
	Control	30.97	0.48	31.26	0.42	0.31	0.28	0.28
Sig.**		0.78		< 0.001		––––	< 0.001	

^*^paired t-test, **independent samples t-test

The mean score of outcome expectations in the intervention group increased from 46.14 ± 6.42 at baseline to 57.02 ± 4.53 three months after the intervention. (Paired t-test: t = -18.61, df = 49, *p* < 0.001). However, no significant change was observed within the control group after the intervention. Furthermore, the independent samples t-test showed that the mean changes in outcome expectancy scores were significantly different between the intervention group (10.88 ± 4.13) and the control group (0.34 ± 7.42) three months after the intervention (Table 2).

The mean self-efficacy score in the intervention group was 29.54 ± 2.44 before the intervention and 36.70 ± 5.08 after that (paired sample t-test; t = -11.31, df = 49, *p* < 0.001). However, the mean scores of this construct in the control group before [28.57 ± 6.42] and after the intervention (28.59 ± 6.77) were not statistically different (*p* = 0.23). The mean changes in self-efficacy scores between the intervention (7.16 ± 0.63) and control (0.02 ± 0.51) groups were significantly different, as showed by the independent samples t-test (*p* < 0.001) (Table 2).

The results revealed no significant difference between the intervention and control groups concerning the mean score of perceived social support before the intervention (15.62 ± 4.46 vs. 16.02 ± 2.72). After the intervention, the mean social support score was 28.86 ± 2.89 in the intervention group and 16.24 ± 2.89 in the control group. The independent t-test indicated a statistically significant difference between the groups (t = -22.43, df = 97, *p* < 0.001). This test also indicated a significant difference between the two groups regarding the changes in the social support mean score (Table 2).

Table 2 indicates that the mean self-regulation score in the intervention group changed from 30.76 ± 4.34 at baseline to 42.72 ± 3.88 three months after intervention (Paired sample t-test; t = -16.18, df = 49, *p* < 0.001). However, no significant difference was found in the control group regarding the mean score of this construct before (30.97 ± 3.38) and after (31.26 ± 2.99) the intervention. As indicated by the independent t-test, the mean of self-regulation score changes were higher in the intervention group (11.96 ± 5.22) than in the control group (0.28 ± 1.97).

In the intervention group, the mean self-management score increased from 36.06 ± 10.02 before the intervention to 64.94 ± 7.34 three months after the intervention. Also, the paired t-test showed that the difference was statistically significant (t = -17.72, df = 49, *p* < 0.001). However, this was not the case in the control group. Besides, the independent t-test revealed a significant difference between the two groups concerning the changes in the self-management mean score (Table 3).

**Table 3:** Comparison of mean scores of outcome measures within and between intervention and control groups, before and after training program

Variable	Group	Before		After		Sig.*	Difference	
		Mean	SE	Mean	SE		Mean	SE
Self-management	Intervention	36.06	1.41	64.94	1.03	< 0.001	28.88	1.62
	Control	36.71	1.34	36.91	1.30	0.10	0.20	0.12
Sig.**		0.73		< 0.001		––––	< 0.001	
Quality of life	Intervention	40.50	1.01	57.40	0.96	< 0.001	16.90	1.04
	Control	42.75	1.31	43.32	1.35	0.18	0.57	0.42
Sig.**		0.17		< 0.001		––––	< 0.001	
HbA1c	Intervention	8.29	0.14	6.28	0.18	< 0.001	-2.01	0.09
	Control	8.44	0.19	8.13	0.28	0.10	-0.30	0.18
Sig.**		0.54		< 0.001		––––	< 0.001	
Fasting blood sugar	Intervention	193.64	9.26	127.96	3.67	< 0.001	-65.68	1.10
	Control	192.61	9.08	183.02	8.60	0.19	-9.59	1.05
Sig.**		0.93		*p* < 0.001		––––	< 0.001	
Waist circumference	Intervention	103.50	1.55	98.16	1.38	< 0.001	-5.34	0.57
	Control	99.88	1.52	100.90	1.61	0.11	1.02	0.63
Sig.**		0.10		0.20		––––	< 0.001	
Body mass index	Intervention	31.04	0.76	29.58	0.64	< 0.001	-1.45	0.22
	Control	30.11	0.58	30.05	0.58	0.68	-0.05	0.13
Sig.**		0.33		0.59		––––	< 0.001	
Systolic blood pressure	Intervention	122.40	2.21	113.60	1.99	< 0.001	-8.80	1.68
	Control	117.96	2.55	116.53	2.41	0.27	-1.42	1.30
Sig.**		0.19		0.35		––––	< 0.001	
Diastolic blood pressure	Intervention	77.80	1.67	71.50	1.41	< 0.001	-6.30	1.6
	Control	76.02	1.61	73.98	1.60	0.07	-2.04	1.12
Sig.**		0.34		0.44		––––	0.033	

^*^paired t-test, **independent samples t-test

Table 3 shows that the intervention group's mean quality of life score increased from 40.50 ± 7.21 at baseline to 57.40 ± 6.84 three months after the intervention (Paired t-test: t = -16.15, df = 49, *p* < 0.001). However, this was not the case in the control group. Furthermore, the mean change in the quality-of-life score was significantly higher in the intervention group [16.90 ± 7.39] than in the control group (0.57 ± 2.99).

In the intervention group, the mean HbA1c values decreased from 8.29 ± 1.027 before the intervention to 6.28 ± 1.30 three months after the intervention (Paired t-test: t = 20.88, df = 49, *p* < 0.001). Compared to baseline, no significant change in the mean HbA1c value was observed in the control group after the intervention. In addition, the independent t-test showed that the mean change in HbA1c in the intervention group (-2.01 ± 0.68) was higher than in the control group (-0.30 ± 1.31) (Table 3).

In the intervention group, the mean FBS value decreased from 193.64 ± 65.49 at baseline to 127.96 ± 25.99 three months after the intervention (Independent t-test: t = 5.75, df = 97, *p* < 0.001). No significant change was observed in the mean FBS values in the control group after the intervention. The independent t-test also showed that the mean change in FBS values after the intervention was significantly different between the intervention (-65.68 ± 7.78) and the control groups (-9.59 ± 7.37) (Table 3).

Considering the intra-group comparison based on paired t-test, significant differences were found in the intervention group in terms of mean scores of waist circumference (WC), body mass index (BMI), systolic and diastolic blood pressure (SBP & DBP) after the intervention. In this group, the mean WC score decreased from 103.50 ± 11.01 before the intervention to 98.16 ± 9.77 three months after the intervention (t = 9.30, df = 49, *p* < 0.001). In addition, the mean score of BMI significantly decreased from 31.04 ± 5.38 to 29.58 ± 4.55 three months after the intervention (t = 6.43, df = 49, *p* < 0.001). Besides, the mean SBP score decreased from 122.40 ± 15.85 before the intervention to 113.60 ± 14.21 three months after the intervention (t = 5.21, df = 49, *p* < 0.001). Finally, the mean DBP score decreased from 77.80 ± 11.83 before the intervention to 71.50 ± 10.01 three months after the intervention (t = 0.11, df = 49, *p* < 0.001). Despite the slight differences observed in the baseline, the independent samples t-test revealed no significant differences between the two groups in terms of these variables both at baseline and three-month after the intervention. However, the mean changes in the scores of these variables, as shown by the independent samples t-tests, were significantly different between the two groups after the intervention (Table 3).

Regression analysis was performed to determine the extent to which the SCT constructs [independent variables] could explain the variance of quality of life [dependent variable] in the studied patients (Table 4). The results demonstrated that all the constructs, except for self-efficacy, could significantly explain and predict 40% of the variance of quality of life (*R*^*2*^ = 0.476, adjusted *R*^*2*^ = 0.402, F = 6.49, *p* < 0.001). Accordingly, each unit's increase in knowledge, self-management, social support, self-regulation, and outcome expectations improved the mean score of quality of life by 0.249, 0.406, 0.260, 0.280, and 0.401 folds, respectively.

**Table 4:** The results of multivariate regression analysis for predicting quality of life based on the constructs of the social cognitive theory

Predictive variables	B	Beta	T	P
Constant	-14.378	-	-2.713	0.010
Knowledge	0.697	0.249	2.188	0.034
Self-efficacy	0.274	0.166	1.32	0.193
Self-management	0.261	0.406	3.34	0.002
Social support	0.437	0.260	2.27	0.028
Self-regulation	0.396	0.280	2.21	0.032
Outcome expectations	0.718	0.401	3.49	0.001

## Discussion

This study aimed to determine the effect of an SCT-based educational program followed by home visits on self-management behavior, glycemic index, and quality of life among T2DM patients. The findings indicated that multidisciplinary intervention with home visits significantly improved self-management behavior, glycemic index, and quality of life among T2DM patients in the interventional group compared to the control group. Before the intervention, no statistically significant difference was observed between the intervention and control groups concerning the desired variables, showing that the changes in the study's primary outcomes favored the educational intervention.

The findings have revealed a significant increase in the mean scores of SCT constructs, including knowledge, social support, self-efficacy, outcome expectation, and self-regulation among T2DM patients. These findings are consistent with the results of Mazloomi [36], Borhaninejad [16], Bougar [37], Tan. Ming [38], and Schillinger [39]. Borhaninejad et al. reported a significant positive correlation between self-care and T2DM knowledge. Besides, diabetes knowledge predicted 57% of the variance of self-care behaviors (*p* < 0.001) [40].

In addition, the perceived social support of patients was better in the intervention group than in the control group after the educational intervention. Similar results were obtained by Mazloomi [36], Mayberry [41], and Wang [42]. Haidari also studied the effect of the relationship between family support and blood sugar control among older adults with T2DM and reported a significant negative correlation between family support and HbA1C level [43]. Mazloomi et al. explored the predictors of self-care behavior in 200 T2DM patients based on the SCT and revealed an increase in the perceived social support [36]. In the same vein, the results of a study conducted by the American Diabetes Association in 2017 to determine the relationship between family support and glycemic control and medication adherence in adults with Type 2 diabetes indicated that patients with lower family support had weaker control [41]. Madden’s study also demonstrated that the patients who communicate better with their family and friends had better control over their illness, reflecting the perceived social support of an emotional type [44]. Furthermore, Nicklett et al. stated that social support was strongly associated with adherence to diabetes diet therapy and played a crucial role in promoting health [45]. Wang also reported that self-care, medication, and adherence behaviors were much higher in the patients with T2DM who were supported by their families and friends than in those who were not [42].

Furthermore, the participants in the intervention group displayed a significant increase in self-efficacy, which was in line with the results of Borhaninejad [40], Mazlumi [36], and Nouwen [46]. Wichit also conducted a randomized controlled trial on a family-centered self-management program to improve self-efficacy, glycemic control, and quality of life among individuals with T2DM. The results demonstrated significantly better self-efficacy, self-management, outcome expectations, and knowledge of diabetes in the intervention group than in the control group [47]. Chih et al. indicated that adolescents with Type 1 diabetes with high self-efficacy were 1.63 times more likely to achieve glycemic control [48].

The outcome expectation level among the participants had a significant increase in the intervention group as well, which was consistent with the results of the studies performed by Wichit [47], Jalily [49], and Heidari-Soureshjani [12]. Jalily et al. investigated the predictors of nutritional behaviors based on the SCT in pregnant women. They reported that awareness, outcome expectations, outcome expectancies, and self-regulation could effectively help design educational interventions to achieve healthy eating behaviors in pregnant women [49]. Heidari-Soureshjani et al. also investigated 198 women with diabetes in Shahrekord. They demonstrated that following health behaviors [diet and physical activity] directly correlated with outcome expectations, self-efficacy, and self-regulation [12].

As another concept of social cognitive theory, a significant increase was also observed in the self-regulation level among the participants in the intervention group. Although we could find no similar interventional studies based on the SCT in T2DM patients, this finding agreed with those obtained by Cellar [50], Heidari-Soureshjani [43], and Borhaninejad [16]. The research results performed by Peyman et al. showed that educational intervention using self-regulatory strategies increased physical activity and improved blood sugar and body mass index in women with Type 2 diabetes [28]. Furthermore, Cellar emphasized that goal-setting skills had a strong positive effect on self-regulatory behaviors [50].

In the present study, the final outcome of SCT-based intervention on self-management behavior, glycemic index, and quality of life was also positive. Accordingly, the results showed that the change in the mean score of self-management behavior was significant in the intervention group compared to the control group, proving the continued use of the learned skills in the daily life plans of patients. In other words, the educational program based on the SCT followed by home visits concerning self-management behavior was effective in the intervention group. Borhani Nejad et al. disclosed that using a self-care education program based on the SCT promoted knowledge, self-efficacy, social support, outcome expectations, outcome values, and self-regulation among T2DM patients and increased their self-care [16].

The improved quality of life of the participants in the intervention group was another finding of the present study after the educational intervention. This finding was consistent with [36] and [51]. A study in China examined the effect of the nurses working in home-visit programs on disabled patients and their caregivers and revealed a significant improvement in the intervention group’s social, physical, mental, and life satisfaction after the intervention [52]. Authors in examined thirty-four samples in Turkey during six months of home visits. An improvement was reported in self-care among T2DM patients in the intervention group [53]. In another study assessing the effect of home visits on the quality of life of children and adolescents with Type 1 diabetes, the results indicated that home visits and training positively affected the self-care and quality of life parameters [54].

A comparison of HbA1C and FBS means before and three months after the intervention revealed a significant decrease in the intervention group compared to the control group. In addition, the means of SBP and DBP decreased in the intervention group. However, there was no significant difference between the two groups regarding the means of systolic and diastolic blood pressure. These findings were consistent with those of the studies performed by Chrvala [55], Steinsbekk [51], and Hosseini [56], indicating the prominent effect of education on HbA1C FBS and blood pressure. Systematic reviews by Chrvala et al. [55] and Steinsbekk [51] have also confirmed the positive effect of self-management training on reducing HbA1C.

In the present study, no significant difference was found in the two groups’ mean scores of anthropometric indices [body mass index and waist circumference] before and after the intervention. However, these scores were significantly reduced in the intervention group three months after the intervention. Similar results were also obtained by Ramli [57], Schwedes [58], Steinsbekk [51] and Han [25]. A systematic review and meta-analysis by Steinsbekk also indicated that self-management training significantly improved body weight in group-based diabetes self-management education compared to routine treatment and care among people with T2DM. However, no statistically significant difference was observed between the intervention [self-management training] and control [routine care] groups with respect to body mass index, blood pressure, and blood lipids [51]. In addition, the results of the systematic review by Cortez revealed the effectiveness of empowerment programs in improving HbA1C, metabolic indices, and diastolic blood pressure. However, no significant changes were observed in anthropometric variables [body mass index and waist circumference] and systolic blood pressure in the reviewed studies [59]. Furthermore, Ramli’s study in Brazil showed that empowerment and self-management interventions effectively improved clinical outcomes. In addition to achieving primary results [HbA1C less than 6.7%], patients achieved improvement in secondary outcomes [blood pressure, fat profile, body mass index, and waist circumference] [57], which was not in agreement with the present study findings.

### Strengths and weaknesses of the study

This is the first multi-component intervention with a social cognitive theoretical framework that applies various theoretical and practical methods, participatory learning techniques, and home visits to promote self-management among T2DM patients. Experimental and evidence-based design and evaluation of multiple behavioral and health consequences were also among the strengths of this study. The limitations were as follows: The impact assessment was short-term and, consequently, long-term evaluation is recommended to assess the survival of outcomes. Also, the findings may not be generalized to other populations with different ethnic and socioeconomic characteristics. Moreover, self-reporting tools may have led to social desirability bias in the results. Although the HbA1c levels and FBS have been measured as objective proxy measures to solve this problem in this study, it is suggested that future studies use anthropometric indicators. The study also suffers from relatively small sample size and a lack of power analysis. Removing these three limitations will add to the richness of future research in this area.

## Conclusion

The findings in this study suggest that some changes should be made in research and practice [service delivery] approaches regarding diabetes prevention and control. Changes should be made in routine patient education and care methods, comprehensive behavior change theories, interactive and client-centered approaches [33], and family involvement and support. As one of the critical components of comprehensive interventions, home visits are also strongly recommended. Furthermore, it is strongly recommended that further studies using socio-ecological theoretical frameworks and planning for long-term evaluation are warranted.

## Data Availability

The datasets used and/or analyzed during the current study are available from the corresponding author on reasonable request.
